# Talks with Dr. Mandeville

**Published:** 1892-05

**Authors:** 


### HALL'S JOURNAL OF HEALTH

TRUTH DEMANDS NO SACRIFICE; ERROR CAN MAKE NONE.

Vol. 39. MAY, 1892. No. 5.

### TALKS WITH DR. MANDEVILLE.

No. 4.

Q. Well, Doctor, are you prepared to continue your remarks on the nervous system?

A. Yes, I am quite ready. I believe we left off with a message to the brain. Well, if you put one end of a wire in connection with one pole of a battery, and the other end in connection with the other pole, the electric current will be sent along the wire, which will be exactly like the current which is sent along the nerve. The wire is capable of transmitting a message; but we happen to know that that is not the sort of message which is carried along the nerve when the hot spoon is put upon the hand, causing it to jump. We know it in this way: if you cut open your arm so as to expose the nerve, and tie a string around it, you will find that the message which the nerve naturally communicates to the brain will not be interrupted in its passage, whereas if a string be tied tightly around my arm it will have the same effect as if my arm were paralyzed or the nerve severed at the point of binding, thus proving that the message which goes along the nerve is not the same as that which goes along the telegraph wire, and yet it is something very like it.

Now, we are able to explain with a great degree of accuracy, what it is exactly that goes along the nerve. If you arrange upon a table a series of ordinary playing cards, bent in the middle to make them stand, you will observe that on tapping one of them, the whole row will fall as one, in rapid succession. When a message has been sent along a nerve there is something else which happens, and that is that the blood which is always running around the nerve in exceeding minute pipes or vessels, contrives to build the nerve up again as it was before. Now, what happens when the hot spoon is put against your hand? The hot spoon gives a little jerk to the ends of certain white threads which come out very near to the surface all over your body. You will observe that something travels along, but it is not any material substance. It is the same state of feeling which travels along the cards from one end to the other, because each of them gives the other a little hint as it falls over. Nerves are made up of a number of separate things which are as nearly alike as may be. These are what are called "molecules." Molecules are exceedingly small particles of matter which can go about by themselves. Whenever any kind of matter is reduced into a gaseous state, as water boiled and converted into steam, the molecules of which water is composed, move mostly in straight lines, but turn each other round when they come in contact, precisely as people in dancing Sir Rodger de Coverley dance up in straight lines and turn each other when they meet in the middle. In the nervous substance, those molecules remain as nearly as possible in the same condition—they no doubt oscillate a great deal on each side of their positions, but still they do not travel around about each other as the molecules of air do in a room. The nervous substances are made up of molecules laid along in a row, and all those molecules are little machines which are almost exactly alike.

How do we know that they are little machines? There are two things which show that the separate parts which make up the nervous thread are to be regarded as little machines—because they have two properties which are common to all the machines we know of. One of these properties is known as the law of the conservation of energy. Those are long words, but they just mean that you cannot get any more work out of a machine than is in it. Suppose for example you lift a weight up to a certain distance; you have done a certain amount of work, which is reckoned in foot-pounds—that is, you must measure up the distance you have lifted the weight in feet, and measure the weight in pounds, and multiply the two numbers together, and that will give you the amount of work you have done. Now this energy you have used will always do the same amount of work again. In order to make a steam engine work, you have to supply it with a certain amount of coal; that coal represents work which the sun has done in past ages, and the heat can be brought out by combustion, and a certain portion of heat can be brought out to work the engine, only a certain amount, though, because there is a certain portion of it which must necessarily be wasted. But it is only by the imperfection of the machinery that we lose any portion of the power, and the rule applies to all cases where molecules exist. Whenever we can test cases where molecules are concerned we find the rule to hold good—that they work just the same as other machines, never doing any thing for nothing, and always doing exactly as much as they are paid to do. That is one of the reasons why we are right in saying that those small particles of which the nerve fibre is built up are little machines.

But there is another reason. There is a very general law which applies to all machines whatever, and to all mechanical actions, and that is that the change in the motion of any thing depends upon its position and relation to other things. If a body is moving by itself in the universe without any thing else to disturb it, it would go on moving in that way without any change at all. Any change which takes place in the motion of that body implies that there is something else moving in the universe. Now, there is a certain change taking place in the molecules, which shows that there is a change taking place in parts of them. These are motions of vibration, just like the motions of vibration of a tuning fork when it is struck. These motions of vibration in molecules give rise to what is called light and heat, which are actually the motions of something between thê molecules and ourselves. By means of the phenomena of light and heat we are able to find out what is the exact character of the motion of vibration of the molecules, and we find that although every molecule has a great number of ways of vibrating, just as a plate of glass has a number of ways of vibrating, according to the way in which it is struck, as with the violin bow, yet each of these vibrations taken separately, follows a perfect definite law, by which we can show that the change in its motion at any instant depends upon the relation of the molecules to one another. For those two reasons we have a right to consider that the molecules, the small parts of which matter is built up, are little machines.

[Subject continued in our next issue.]

